# Supplementary material for: A Theoretical Framework to Quantify the Tradeoff Between Individual and Population Benefits of Expanded Antibiotic Use
Source: Bull Math Biol. 2025 Apr 30;87(6):68. doi: 10.1007/s11538-025-01432-2 (PMC12043784; doi:10.1007/s11538-025-01432-2)
Supplement: Supplementary file 1 — (pdf 242 KB) [file 11538_2025_1432_MOESM1_ESM.pdf]

Supplementary Material

Proportion of Exposed Becoming each Infected Type

Here we further describe the pathways for individuals becoming infected after exposure. The five proportions described in the main text $(p_A, p_{MT}, p_{MU}, p_{ST}, p_{SU})$ can be broken down into more measurable decision-related values:

- ϵ_A = proportion of individuals exposed that are asymptomatic
- ϵ_S = proportion of all symptomatic individuals that have severe symptoms ($1-\epsilon_S$ = moderate symptoms)
- $\epsilon_{M_{sy}T}$ = proportion of moderately symptomatic individuals who seek treatment
- $\epsilon_{S_{sy}T}$ = proportion of severely symptomatic individuals who seek treatment

These allow the proportions to sum to 1 and the following equations describe the structure illustrated in Figure S1.

$$
\begin{aligned}
p_A &= \epsilon_A \\
p_{MT} &= (1-\epsilon_A)(1-\epsilon_S)(\epsilon_{M_{sy}T}) \\
p_{MU} &= (1-\epsilon_A)(1-\epsilon_S)(1-\epsilon_{M_{sy}T}) \\
p_{ST} &= (1-\epsilon_A)(\epsilon_S)(\epsilon_{S_{sy}T}) \\
p_{SU} &= (1-\epsilon_A)(\epsilon_S)(1-\epsilon_{S_{sy}T})
\end{aligned}
$$

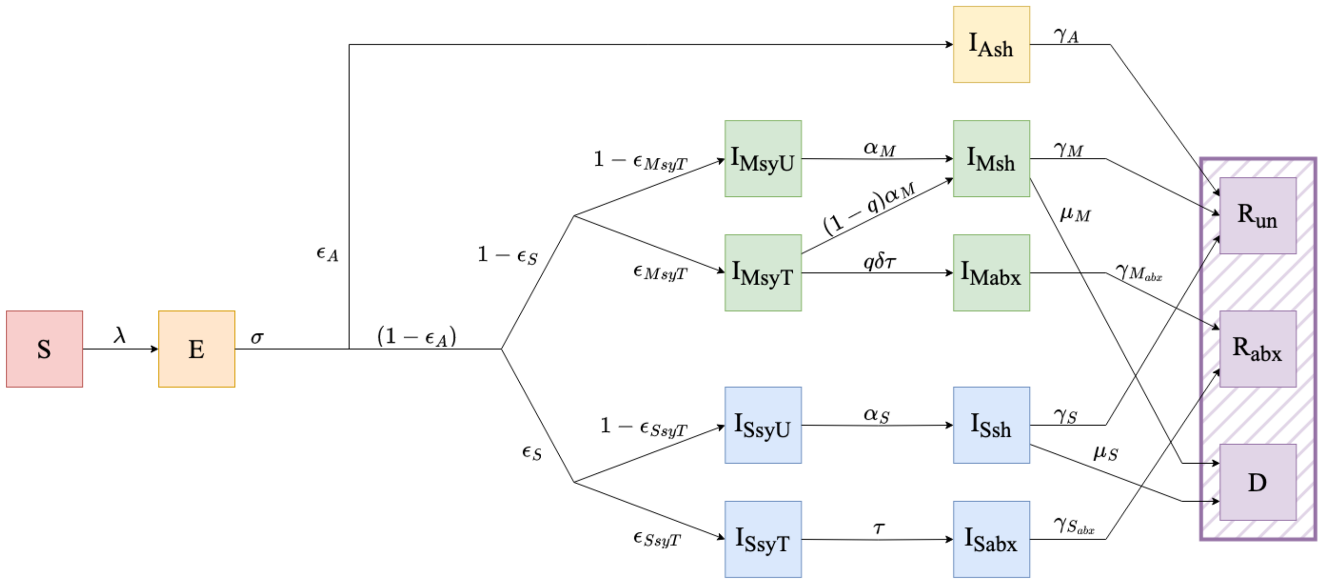

Figure S1: Model diagram with detailed parameterization of pathways between exposure and infection.

Model Equations

$$
\begin{aligned}
\frac{dS}{dt} &= -\lambda S \\
\frac{dE}{dt} &= \lambda S - \sigma E \\
\frac{dI_{A_{sh}}}{dt} &= \sigma p_A E - \gamma_A I_{A_{sh}} \\
\frac{dI_{M_{syU}}}{dt} &= \sigma p_{MU} E - \alpha_M I_{M_{syU}} & \frac{dI_{S_{syU}}}{dt} &= \sigma p_{SU} E - \alpha_S I_{S_{syU}} \\
\frac{dI_{M_{syT}}}{dt} &= \sigma p_{MT} E - ((1-q)\alpha_M + q\delta\theta) I_{M_{syT}} & \frac{dI_{S_{syT}}}{dt} &= \sigma p_{ST} E - \theta I_{S_{syT}} \\
\frac{dI_{M_{sh}}}{dt} &= \alpha_M I_{M_{syU}} + (1-q)\alpha_M I_{M_{syT}} - (\gamma_M + \mu_M) I_{M_{sh}} & \frac{dI_{S_{sh}}}{dt} &= \alpha_S I_{S_{syU}} - (\gamma_S + \mu_S) I_{S_{sh}} \\
\frac{dI_{M_{abx}}}{dt} &= q\delta\theta I_{M_{syT}} - \gamma_{M_{abx}} I_{M_{abx}} & \frac{dI_{S_{abx}}}{dt} &= \theta I_{S_{syT}} - \gamma_{S_{abx}} I_{S_{abx}} \\
\frac{dR}{dt} &= \gamma_A I_{A_{sh}} + \gamma_M I_{M_{sh}} + \gamma_S I_{S_{sh}} & \frac{dR_{abx}}{dt} &= \gamma_{M_{abx}} I_{M_{abx}} + \gamma_{S_{abx}} I_{S_{abx}} \\
\frac{dD}{dt} &= \mu_M I_{M_{sh}} + \mu_S I_{S_{sh}}
\end{aligned}
\tag{1}
$$

Parameter values

Relative Infectiousness Modifiers

The values of ν_A and ν_M are not well characterized in the literature and would benefit from additional clinical research. For the purposes of this study, we find feasible values for these parameters ($\nu_A = 0.250$, $\nu_M = 0.600$) through a combination of literature review and expert elicitation. We know that severely dehydrated cholera patients have the longest duration of diarrhea symptoms (with and without antibiotic treatment), and the longest duration of culture positivity (with and without antibiotics)[30]. Counter-intuitively, the least dehydrated cholera patients with diarrhea had the second longest duration of diarrhea symptoms[30]. We assume that more days of diarrhea with less dehydration is indicative of lower volumes of stool. We further assume that lower volumes of stool equates to less bacteria released into the community, (*i.e.*, less transmission). Finally, we assume that an absence of symptoms leads to better stool management (*e.g.*, more likely to safely dispose of stool in covered pit latrine), further decreasing transmission for asymptomatic cholera patients compared to severely symptomatic cholera patients.

Similarly, ν_{abx} is not well characterized in the literature and would benefit from additional clinical research. For the purposes of this study, we find a feasible value for this parameter ($\nu_{abx} = 0.500$) also through literature review and expert elicitation. It has been shown that among cholera patients treated with antibiotics, the proportion of patients testing positive by culture decreases rapidly over the first 2 days after treatment, with no culture positives individuals by day 4[22]. Similarly, it has been found that cholera patients were culture positive an average of 2.6 days after antibiotics[30], whereas other studies documented an average of 1 day of stool culture positivity following antibiotic treatment[54]. While these estimates speak to the rate at which cholera patients treated with antibiotics recover, recovery is not a binary process where shedding is immediately halted. Therefore we opted for a conservative estimate of half transmission to encompass the time spent transitioning from the start of treatment to recover

Final Size Equation Derivation

We rewrite here the relevant equations of the system and give them individual labels for reference.

$$\text{(f.1)} \quad \frac{dS}{dt} = -\lambda S$$

$$\text{(f.2)} \quad \frac{dE}{dt} = \lambda S - \sigma E$$

$$\text{(f.3)} \quad \frac{dI_{M_{sy}U}}{dt} = \sigma p_{MU} E - \alpha_M I_{M_{sy}U}$$

$$\text{(f.4)} \quad \frac{dI_{M_{sy}T}}{dt} = \sigma p_{MT} E - ((1-q)\alpha_M + q\delta\theta) I_{M_{sy}T}$$

$$\text{(f.5)} \quad \frac{dI_{M_{sh}}}{dt} = \alpha_M I_{M_{sy}U} + (1-q)\alpha_M I_{M_{sy}T} - (\gamma_M + \mu_M) I_{M_{sh}}$$

$$\text{(f.6)} \quad \frac{dI_{M_{abx}}}{dt} = q\delta\theta I_{M_{sy}T} - \gamma_{M_{abx}} I_{M_{abx}}$$

$$\text{(f.7)} \quad \frac{dI_{A_{sh}}}{dt} = \sigma p_A E - \gamma_A I_{A_{sh}}$$

$$\text{(f.8)} \quad \frac{dI_{S_{sy}U}}{dt} = \sigma p_{SU} E - \alpha_S I_{S_{sy}U}$$

$$\text{(f.9)} \quad \frac{dI_{S_{sy}T}}{dt} = \sigma p_{ST} E - \theta I_{S_{sy}T}$$

$$\text{(f.10)} \quad \frac{dI_{S_{sh}}}{dt} = \alpha_S I_{S_{sy}U} - (\gamma_S + \mu_S) I_{S_{sh}}$$

$$\text{(f.11)} \quad \frac{dI_{S_{abx}}}{dt} = \theta I_{S_{sy}T} - \gamma_{S_{abx}} I_{S_{abx}}$$

$$\text{(f.12)} \quad \frac{1}{S}\frac{dS}{dt} = -\lambda = -\beta[(I_{S_{sy}U} + I_{S_{sy}T}) + \nu_{sh} I_{S_{sh}} + \nu_A I_{A_{sh}} + \nu_M (I_{M_{sy}U} + I_{M_{sy}T}) + \nu_{sh}\nu_M I_{M_{sh}} + \nu_M \nu_{abx} I_{M_{abx}}]$$

$$\text{(f.13)} \quad \frac{dR_{un}}{dt} = \gamma_A I_{A_{sh}} + \gamma_M I_{M_{sh}} + \gamma_S I_{S_{sh}}$$

$$\text{(f.14)} \quad \frac{dR_{abx}}{dt} = \gamma_{M_{abx}} I_{M_{abx}} + \gamma_{S_{abx}} I_{S_{abx}}$$

$$\text{(f.15)} \quad \frac{dD}{dt} = \mu_M I_{M_{sh}} + \mu_S I_{S_{sh}}$$

$$\text{(f.16)} \quad N = S_\infty + R_{un\infty} + R_{abx\infty} + D$$

The following method avoids using (f.1) and (f.2) to avoid complications from the force of infection λ.

To find the final size, we integrate our equation over the time interval from 0 to ∞[32]. We start by setting the infected state equation integrals equal to 0 to simplify each equation in terms of $\int Edt$.

From setting the integral of (f.3) = 0:

$$\sigma p_{MU} \int_0^\infty Edt = \alpha_M \int_0^\infty I_{M_{sy}U} dt \Rightarrow \boxed{\int_0^\infty I_{M_{sy}U} dt = \frac{\sigma p_{MU}}{\alpha_M} \int_0^\infty Edt}$$

From setting the integral of (f.4) = 0:

$$\sigma p_{MT}\int_0^\infty Edt = ((1-q)\alpha_M + q\delta\theta)\int_0^\infty I_{M_{sy}T}dt \Rightarrow \boxed{\int_0^\infty I_{M_{sy}T}dt = \frac{\sigma p_{MT}}{(1-q)\alpha_M + q\delta\theta}\int_0^\infty Edt}$$

From setting the integral of (f.5) = 0:

$$\alpha_M\int_0^\infty I_{M_{sy}U}dt + (1-q)\alpha_M\int_0^\infty I_{M_{sy}T}dt = (\gamma_M+\mu_M)\int_0^\infty I_{M_{sh}}dt$$
$$\Rightarrow \int_0^\infty I_{M_{sh}}dt = \frac{\alpha_M}{\gamma_M+\mu_M}\left((1-q)\int_0^\infty I_{M_{sy}U}dt + \int_0^\infty I_{M_{sy}T}dt\right)$$
$$\Rightarrow \boxed{\int_0^\infty I_{M_{sh}}dt = \frac{\alpha_M}{\gamma_M+\mu_M}\left(\frac{\sigma p_{MU}}{\alpha_M} + \frac{(1-q)\sigma p_{MT}}{(1-q)\alpha_M + q\delta\theta}\right)\int_0^\infty Edt}$$

From setting the integral of (f.6) = 0:

$$q\delta\theta\int_0^\infty I_{M_{sy}T}dt = \gamma_{M_{abx}}\int_0^\infty I_{M_{abx}}dt \Rightarrow \boxed{\int_0^\infty I_{M_{abx}}dt = \frac{q\delta\theta}{\gamma_{M_{abx}}}\left(\frac{\sigma p_{MT}}{(1-q)\alpha_M + q\delta\theta}\right)\int_0^\infty Edt}$$

From setting the integral of (f.7) = 0:

$$\sigma p_A\int_0^\infty Edt = \gamma_A\int_0^\infty I_{A_{sh}}dt \Rightarrow \boxed{\int_0^\infty I_{A_{sh}}dt = \frac{\sigma p_A}{\gamma_A}\int_0^\infty Edt}$$

From setting the integral of (f.8) = 0:

$$\sigma p_{SU}\int_0^\infty Edt = \alpha_S\int_0^\infty I_{S_{sy}U}dt \Rightarrow \boxed{\int_0^\infty I_{S_{sy}U}dt = \frac{\sigma p_{SU}}{\alpha_S}\int_0^\infty Edt}$$

From setting the integral of (f.9) = 0:

$$\sigma p_{ST}\int_0^\infty Edt = \theta\int_0^\infty I_{S_{sy}T}dt \Rightarrow \boxed{\int_0^\infty I_{S_{sy}T}dt = \frac{\sigma p_{ST}}{\theta}\int_0^\infty Edt}$$

From setting the integral of (f.10) = 0:

$$\alpha_S\int_0^\infty I_{S_{sy}U}dt = (\gamma_S+\mu_S)\int_0^\infty I_{S_{sh}}dt \Rightarrow \boxed{\int_0^\infty I_{S_{sh}}dt = \frac{\sigma p_{SU}}{\gamma_S+\mu_S}\int_0^\infty Edt}$$

From setting the integral of (f.11) = 0:

$$\theta\int_0^\infty I_{S_{sy}t}dt = \gamma_{S_{abx}}\int_0^\infty I_{S_{abx}}dt \Rightarrow \boxed{\int_0^\infty I_{S_{abx}}dt = \frac{\sigma p_{ST}}{\gamma_{S_{abx}}}\int_0^\infty Edt}$$

We then find the final sizes of the desired states in terms of $\int Edt$.

From the integral of (f.13):

$$\int_0^\infty \frac{dR_{un}}{dt}dt = R_{un\infty} - R_0 = R_{un\infty} = \gamma_A \int_0^\infty I_{A_{sh}}dt + \gamma_M \int_0^\infty I_{M_{sh}}dt + \gamma_S \int_0^\infty I_{S_{sh}}dt$$

$$\Rightarrow \boxed{R_{un\infty} = \frac{\alpha_M \gamma_M}{\gamma_M + \mu_M}\left(\frac{\sigma p_{MU}}{\alpha_M} + \frac{(1-q)\sigma p_{MT}}{(1-q)\alpha_M + q\delta\theta}\right)\int_0^\infty Edt + \sigma p_A \int_0^\infty Edt + \frac{\sigma p_{SU}\gamma_S}{\gamma_S + \mu_S}\int_0^\infty Edt}$$

From the integral of (f.14):

$$\int_0^\infty \frac{dR_{abx}}{dt}dt = R_{abx\infty} = \gamma_{M_{abx}} \int_0^\infty I_{M_{abx}}dt + \gamma_{S_{abx}} \int_0^\infty I_{S_{abx}}dt$$

$$\Rightarrow \boxed{R_{abx\infty} = q\delta\theta\left(\frac{\sigma p_{MT}}{(1-q)\alpha_M + q\delta\theta}\right)\int_0^\infty Edt + \sigma p_{ST}\int_0^\infty Edt}$$

From the integral of (f.15):

$$\int_0^\infty \frac{dD}{dt}dt = D_\infty = \mu_M \int_0^\infty I_{M_{sh}}dt + \mu_S \int_0^\infty I_{S_{sh}}dt$$

$$\Rightarrow \boxed{D_\infty = \frac{\alpha_M \mu_M}{\gamma_M + \mu_M}\left(\frac{\sigma p_{MU}}{\alpha_M} + \frac{(1-q)\sigma p_{MT}}{(1-q)\alpha_M + q\delta\theta}\right)\int_0^\infty Edt + \frac{\sigma p_{SU}\mu_S}{\gamma_S + \mu_S}\int_0^\infty Edt}$$

From here we want to find the $\int Edt$ to plug in above. We will use the trick of dividing both sides by S. From there we integrate and plug in the integrals from the infected states found above. (f.12) then integrates to solve for $\int Edt$.

$$\begin{aligned}
\frac{1}{S}\frac{dS}{dt} &= -\lambda \\
\int_0^\infty \frac{dS}{S} &= \int_0^\infty -\beta\left[(I_{S_{syU}} + I_{S_{syT}}) + \nu_{sh} I_{S_{sh}} + \nu_A I_{A_{sh}}\right. \\
&\qquad \left. + \nu_M (I_{M_{syU}} + I_{M_{syT}}) + \nu_{sh}\nu_M I_{M_{sh}} + \nu_M \nu_{abx} I_{M_{abx}}\right] dt \\
-\frac{1}{\beta}\left(\ln(S_\infty) - \ln(S_0)\right) &= \frac{\sigma p_{SU}}{\alpha_S}\int_0^\infty Edt + \frac{\sigma p_{ST}}{\theta}\int_0^\infty Edt + \nu_{sh}\frac{\sigma p_{SU}}{\gamma_S + \mu_S}\int_0^\infty Edt \\
&\quad + \nu_A \frac{\sigma p_A}{\gamma_A}\int_0^\infty Edt \\
&\quad + \nu_M \frac{\sigma p_{MU}}{\alpha_M}\int_0^\infty Edt + \nu_M \frac{\sigma p_{MT}}{(1-q)\alpha_M + q\delta\theta}\int_0^\infty Edt \\
&\quad + \nu_{sh}\nu_M \frac{\alpha_M}{\gamma_M + \mu_M}\left(\frac{\sigma p_{MU}}{\alpha_M} + \frac{(1-q)\sigma p_{MT}}{(1-q)\alpha_M + q\delta\theta}\right)\int_0^\infty Edt \\
&\quad + \nu_M \nu_{abx}\frac{q\delta\theta}{\gamma_{M_{abx}}}\left(\frac{\sigma p_{MT}}{(1-q)\alpha_M + q\delta\theta}\right)\int_0^\infty Edt \\
\Rightarrow &\boxed{\int_0^\infty Edt = -\frac{1}{\beta}\left(\ln(S_\infty) - \ln(S_0)\right)/K}
\end{aligned}$$

where

$$\begin{aligned}
K &= \frac{\sigma p_{SU}}{\alpha_S} + \frac{\sigma p_{ST}}{\theta} + \nu_{sh}\frac{\sigma p_{SU}}{\gamma_S+\mu_S} + \nu_A\frac{\sigma p_A}{\gamma_A} + \nu_M\frac{\sigma p_{MU}}{\alpha_M} \\
&\quad + \nu_M\frac{\sigma p_{MT}}{(1-q)\alpha_M+q\delta\theta} + \nu_{sh}\nu_M\frac{\alpha_M}{\gamma_M+\mu_M}\left(\frac{\sigma p_{MU}}{\alpha_M} + \frac{(1-q)\sigma p_{MT}}{(1-q)\alpha_M+q\delta\theta}\right) \\
&\quad + \nu_M\nu_{abx}\frac{q\delta\theta}{\gamma_{M_{abx}}}\left(\frac{\sigma p_{MT}}{(1-q)\alpha_M+q\delta\theta}\right) \\
&= \frac{\mathcal{R}}{\beta N}
\end{aligned}$$

Therefore

$$\int_0^\infty E dt = \frac{N}{\mathcal{R}}(\ln(S_0) - \ln(S_\infty))$$

From here we use (f.16) to put all of the pieces together:

$$\begin{aligned}
N &= S_\infty + R_{un\infty} + R_{abx\infty} + D \\
S_\infty - N &= -\int_0^\infty E dt\left[\frac{\alpha_M\gamma_M}{\gamma_M+\mu_M}\left(\frac{\sigma p_{MU}}{\alpha_M} + \frac{(1-q)\sigma p_{MT}}{(1-q)\alpha_M+q\delta\theta}\right) + \sigma p_A + \frac{\sigma p_{SU}\gamma_S}{\gamma_S+\mu_S}\right. \\
&\qquad + q\delta\theta\left(\frac{\sigma p_{MT}}{(1-q)\alpha_M+q\delta\theta}\right) + \sigma p_{ST} \\
&\qquad \left. + \frac{\alpha_M\mu_M}{\gamma_M+\mu_M}\left(\frac{\sigma p_{MU}}{\alpha_M} + \frac{\sigma p_{MT}}{(1-q)\alpha_M+q\delta\theta}\right) + \frac{\sigma p_{SU}\mu_S}{\gamma_S+\mu_S}\right] \\
S_\infty - N &= -\frac{N}{\mathcal{R}}(\ln(S_0) - \ln(S_\infty))C \\
S_\infty - N &= -\frac{N}{\mathcal{R}}(\ln(S_0) - \ln(S_\infty))
\end{aligned}$$

where

$$\begin{aligned}
C &= \frac{\alpha_M\gamma_M}{\gamma_M+\mu_M}\left(\frac{\sigma p_{MU}}{\alpha_M} + \frac{(1-q)\sigma p_{MT}}{(1-q)\alpha_M+q\delta\theta}\right) + \sigma p_A + \frac{\sigma p_{SU}\gamma_S}{\gamma_S+\mu_S} \\
&\quad + q\delta\theta\left(\frac{\sigma p_{MT}}{(1-q)\alpha_M+q\delta\theta}\right) + \sigma p_{ST} \\
&\quad + \frac{\alpha_M\mu_M}{\gamma_M+\mu_M}\left(\frac{\sigma p_{MU}}{\alpha_M} + \frac{(1-q)\sigma p_{MT}}{(1-q)\alpha_M+q\delta\theta}\right) + \frac{\sigma p_{SU}\mu_S}{\gamma_S+\mu_S} \\
&= p_{SU} + p_{ST} + p_A + p_{MU} + p_{MT} \\
&= 1
\end{aligned}$$

We continue to solve for s_∞, where $s_\infty = S_\infty/N$.

$$\begin{aligned}
(S_\infty - N)\frac{\mathcal{R}}{N} &= \ln(S_\infty) - \ln(N) \\
\frac{S\infty}{N} &= e^{\frac{\mathcal{R}}{N}(S_\infty - N)} \\
\boxed{s_\infty = e^{\mathcal{R}(s_\infty - 1)}}
\end{aligned}$$

Importantly, this is the standard result for typical SIR and SEIR models as well.

So if we have

$$\int_0^\infty E dt = \frac{N}{\mathcal{R}}(\ln(S_0) - \ln(S_\infty))$$

and

$$s_\infty = e^{\mathcal{R}(s_\infty - 1)} \tag{2}$$

then we can get equations for the final sizes for R_{un}, R_{abx}, and D by plugging these into the equations for $R_{un\infty}$, $R_{abx\infty}$, and D_∞ above after solving for s_∞ numerically.

$$R_{un\infty} = \left(\frac{\alpha_M\gamma_M}{\gamma_M + \mu_M}\left(\frac{\sigma p_{MU}}{\alpha_M} + \frac{\sigma p_{MT}}{(1-q)\alpha_M + q\delta\theta}\right) + \sigma p_A + \frac{\sigma p_{SU}\gamma_S}{\gamma_S + \mu_S}\right)\frac{N}{\mathcal{R}}\left(\ln(N) - \ln(s_\infty N)\right) \tag{3}$$

$$R_{abx\infty} = \left(q\delta\theta\left(\frac{\sigma p_{MT}}{(1-q)\alpha_M + q\delta\theta}\right) + \sigma p_{ST}\right)\frac{N}{\mathcal{R}}\left(\ln(N) - \ln(s_\infty N)\right) \tag{4}$$

$$D_\infty = \left(\frac{\alpha_M\mu_M}{\gamma_M + \mu_M}\left(\frac{\sigma p_{MU}}{\alpha_M} + \frac{\sigma p_{MT}}{(1-q)\alpha_M + q\delta\theta}\right) + \frac{\sigma p_{SU}\mu_S}{\gamma_S + \mu_S}\right)\frac{N}{\mathcal{R}}\left(\ln(N) - \ln(s_\infty N)\right) \tag{5}$$

To study the proportions of individuals in each class, we divide out N and will call the proportional final sizes $r_{un\infty} = R_{un\infty}/N$, $r_{abx\infty} = R_{abx\infty}/N$, and $d_\infty = D_\infty/N$.

$$r_{un\infty} = \left(\frac{\alpha_M\gamma_M}{\gamma_M + \mu_M}\left(\frac{\sigma p_{MU}}{\alpha_M} + \frac{\sigma p_{MT}}{(1-q)\alpha_M + q\delta\theta}\right) + \sigma p_A + \frac{\sigma p_{SU}\gamma_S}{\gamma_S + \mu_S}\right)\frac{1}{\mathcal{R}}\left(-\ln(s_\infty)\right) \tag{6}$$

$$r_{abx\infty} = \left(q\delta\theta\left(\frac{\sigma p_{MT}}{(1-q)\alpha_M + q\delta\theta}\right) + \sigma p_{ST}\right)\frac{1}{\mathcal{R}}\left(-\ln(s_\infty)\right) \tag{7}$$

$$d_\infty = \left(\frac{\alpha_M\mu_M}{\gamma_M + \mu_M}\left(\frac{\sigma p_{MU}}{\alpha_M} + \frac{\sigma p_{MT}}{(1-q)\alpha_M + q\delta\theta}\right) + \frac{\sigma p_{SU}\mu_S}{\gamma_S + \mu_S}\right)\frac{1}{\mathcal{R}}\left(-\ln(s_\infty)\right) \tag{8}$$

High and Low Parameters

We calculate our threshold values for the OPT and DUT for the low and high values of the parameter ranges in Table 3. We find $\mathcal{R}_{dut} = 1.59$ and $\mathcal{R}_{opt} = 1.53$ for the low end of the parameter range and $\mathcal{R}_{dut} = 1.36$ and $\mathcal{R}_{opt} = 1.24$ for the high end of the parameter range (Figure 4).
